# Positioning an Agenda on a Loving Pedagogy in Second Language Acquisition: Conceptualization, Practice, and Research

**DOI:** 10.3389/fpsyg.2022.894190

**Published:** 2022-05-20

**Authors:** Yongliang Wang, Ali Derakhshan, Ziwen Pan

**Affiliations:** ^1^School of College English Teaching and Research, Henan University, Kaifeng, China; ^2^Department of English Language and Literature/Faculty of Humanities and Social Sciences, Golestan University, Gorgan, Iran; ^3^School of Foreign Languages, Henan University, Kaifeng, China

**Keywords:** second language acquisition, loving pedagogy, positive psychology, love, language education

## Abstract

Second/foreign language teaching has been found as one of the most emotional professions worldwide. To generate optimal academic outcomes and run an effective education, teachers and students’ emotions and feelings must be positively cared for. Given the significance of emotions in L2 education, many studies have followed positive psychology (PP) and examined various positive constructs. Nevertheless, love, as a PP variable, has been ignored in education due to its cultural/religious sensitivities. Trying to dispel the myths, recently, a new trend called a “loving pedagogy” has started to find itself a place in second language acquisition (SLA) research and practice. Yet, proposing a model of its application and an agenda for its research has been overlooked by scholars in this domain. Motivated by this lacuna, this research article provided the conceptualization, definitions, research bases, practical models, and implications of a loving pedagogy for SLA practitioners and future researchers.

## Introduction

Undoubtedly, language teaching and learning are both determined by an ocean of inner feelings, emotions, and internal psychological drives (Dewaele and Li, 2020; Sikma, 2021). This signifies the criticality of emotions in education and educational success (Arnold, 1999). The shift of focus from mere linguistic and pedagogical concerns to students’ and teachers’ emotions began decades ago in applied linguistics (Moskovitz, 1978) but flourished in a new trend in psychology known as *positive psychology* (PP, hereafter). PP maintains that humans’ actions and practices are absolutely linked to their emotions and in case they have positive emotionality, their success, well-being, and improvement are more outstanding (MacIntyre et al., 2019). While it endorses the existence and role of negative emotions in second/foreign language education, PP mainly dwells on the power of positive emotions and constructs in bringing about ever-growing desirable outcomes in academia. One of such critical positive emotions that has been limitedly explored and discussed in educational settings, especially L2 education, is “love.” The concept of love, without a shadow of a doubt, is an inseparable part of a human being’s life and profession. It is almost impossible to conceive education without certifying love. Love and education are two intermingled entities, and it can be claimed that an education wherein love has no place is not education at all. The main purpose of love in education is to establish a context oriented toward students’ and teachers’ emotions, care, and respect in order to humanize the process of learning/teaching and generate positive outcomes like increased motivation, engagement, well-being, success, social competence, and the like.

Despite these opportunities and benefits that the concept of love can provide, it has long been an elephant in the room in applied linguistics with many scholars and practitioners being, respectively, afraid of using the term in their studies and classes (Wilkinson and Kaukko, 2020). While the concept of *love* is seemingly simple, it is a pervasive emotion that can influence each and every aspect of language education. It has been mostly ignored in education due to ethical, cultural, and religious concerns in many contexts that see love as a romantic word disregarding the fact that love is really the essence of humans’ living (Buscaglia and Short, 1982). Trying to uproot and ruin these dogmatic conceptions of love, recently, some seminal and respectful attempts have been made by scholars in different settings that resulted in a growing warm acceptance of the term in educational milieus (Loreman, 2011; Grimmer, 2021; Xie and Derakhshan, 2021). These efforts culminated in the introduction to the concepts of *pedagogical love* and *loving pedagogy* in language teaching (Määttä and Uusiautti, 2011; Clingan, 2015; Gidley, 2016; Yin et al., 2019) referring to the care, sensitivity, and empathy that exist among teachers and students regarding their needs, experiences, and development (Yin et al., 2019). The scant literature on this variable reveals that “a pedagogy of love” has the potential to bring about many positive academic outcomes, such as improved motivation, autonomy, agency, well-being, engagement, achievement, self-esteem, critical thinking, academic success, positive interpersonal behaviors, and many more (Grimmer, 2021; Xie and Derakhshan, 2021). An education based on love sets the ground for teachers and students to act efficiently in a more democratic and friendly climate in which the relationship considers both positive and negative emotions (Katz et al., 2008; Lanas and Zembylas, 2015). Such an emotional orientation gives learners a chance to share their inner feelings and establish immediacy and rapport in the class that is the starting point of academic success.

These benefits and opportunities have inspired educators to focus their attention on a loving pedagogy, especially in early education. However, the investigation of this line of inquiry has been limitedly attended to in language education due to its newness and SLA practitioners’ hesitancy toward this topic (Freire, 1996; Barcelos and Coelho, 2016). Another gap is related to the methodological, research, and practical underpinnings by which the concept of “love” can permeate into the educational jargon of L2 educators worldwide. Against these shortcomings, the present review article attempted to present the conceptualization, definitions, research bases, and practical applications of following and implementing a loving pedagogy.

## Background

### The Definition of Love in Education

Owing to its strong attachment to humans’ nature and need for belongingness, providing a universally embraced definition for the concept of love is a daunting task. This is because love means different things in different cultural and professional contexts. Although it seems easy to do so, this complex and pervasive variable is associated with numerous meanings, emotions, affections, and layers in private and occupational settings (Grimmer, 2021). Despite the recent developmental attempts, still most people express love judicially only in private and familial environments (Vincent, 2016a). This has to do with an old-fashioned conceptualization of love regarding it as a soft, feminized word to be avoided in academia as it embodies sentimentality and sexuality among stakeholders. This dogmatic and stubborn view of love being reflected in humans’ life, behavior, needs, and practice is now superseded by “love as both an emotion and an action” (Hooks, 2000, p. 3) that facilitates the expression of professional love among people in the organizational and instructional settings through their caring and respectful interactions and rapports.

As pinpointed by Loreman (2011), love can go beyond the private sphere of the family to make sense in the professional setting, food industry, interpersonal relationships, nature, and many more areas. It has now constructed and established an identity for itself in language teaching referring to teachers’ kindness, affection, empathy, and care toward their students’ feelings, needs, learning, and achievement (Zhao and Li, 2021). This sense of professional love demonstrates that teachers’ and students’ personal and inner emotions and the professional context where they work are no longer separated through exclusive lines. This is in tune with the social nature of human beings which considers various needs and emotions emerging from both personal self and social-professional self. However, educators must err on the side of caution that a pedagogy of love can cause increased academic achievement, engagement, and performance only if it absolutely follows the ethical and professional etiquettes of the cultural context in which it is implemented. In sum, love in education can be defined as teachers’ and educators’ empathy, care, value, and respect for students’ inner states, needs, feelings, and different personality types.

### Conceptualization of a Pedagogy of Love

The earliest trace to the notion of love in education and human development can date back to the early 16th century and the traditional Chinese education philosophy advanced by Mencious, a Chinese thinker, politician, and educationist in the age of the Spring and the Autumn Waring States Period (770–221 BC), who once proposed that “the benevolent loves others” (Ren Zhe Ai Ren in Chinese, Feng, 2012), which literally means those people with perfect virtue have universal love. Likewise, other popular philosophers like Roger Ascham, John Locke, and Martti Haavio highlighted the role of love in education. These scholars dismissed love as an influential learning motivator, the root of good teaching, and a reflector of students’ personalities (Määttä and Uusiautti, 2011; Cousins, 2017). For Freire (1970), education is an act of love. Similarly, Hooks (2003) considered love as the foundation of classroom interactions. Love is also a significant human need as depicted in the hierarchy of needs of Maslow (1954). The concept of love has been historically linked to four Greek terms carrying the same meaning (Nygren, 1953; Herrada, 2013; Aslanian, 2018). They include *agape* (love of mankind), *philia* (love of friends and equals), *eros* (erotic, passionate love), and *storage* (love of parents for children). Yet, love is considered a taboo term to be used in academic contexts and its application is restricted to personal spaces. As pinpointed by Barcelos and Coelho (2016), the reasons for this simplistic view were irrational myths about the preventive impact of love on academic knowledge and learning as well as its damaging role in teachers’ professionalism. For the followers of these views, the teaching atmosphere should be detached and emotionless, and there is no need for caring about students’ feelings. Other than such conceptualizations, previous studies report that due to its strong association with humans’ life and nature, the concept of love has been clarified through different psychological, religious, and philosophical ideas. For example, Sternberg (1986) proposed the first theoretical lens for love in a tripartite theory involving three components of *intimacy*, *passion*, and *decision/commitment*. For him, effective relationships in education happen if all these three elements emerge together. Furthermore, religious perspectives viewed love as a divine property and a gift of God (Zhao and Li, 2021). Likewise, philosophical conceptualizations related love to a quest for beauty.

Despite these historical origins, the expression of love in education remained a sensitive and fearsome act and many educators preferred to use other soft descriptors of love such as passion, care, bond, and affect in order to get away from cultural interpretations of classroom relationship in the academic arena, yet those terms could not convey the same meaning as love, nor did they have its expressive power. These shortcomings triggered an impetus for running some seminal studies by some leading scholars in this domain for whom love and education are two interrelated entities, and all learning/teaching is considered an act of love (Freire, 1996; Hooks, 2003; Loreman, 2011; Määttä and Uusiautti, 2011; Grimmer, 2021; Wang et al., 2021). Now, it is clear that a pedagogy based on love plays a significant role in promoting students’ emotional status, classroom interaction, motivation, social ability, personality, and mental health (Loreman, 2011; Yin et al., 2019). In his seminal research on “love in education,” Loreman (2011) regarded “pedagogy of love” as a positive learning experience for practitioners that encompasses nine psycho-emotional constructs of *passion, kindness, empathy, intimacy, bonding, sacrifice, forgiveness, acceptance*, and *community*.

**Figure 1:**
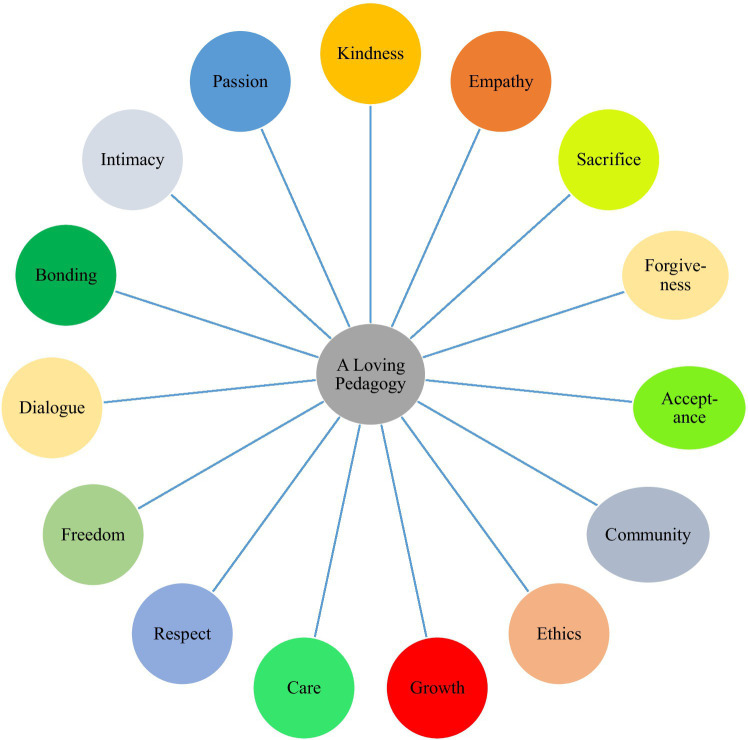
Elements of a pedagogy of love.

In such an academic setting that is sensitive to and cares about students’ emotions, needs, and expectations, a supportive and soft rapport is established between the teacher and the students that, in turn, booms teaching and learning processes. A pedagogy of love integrates these nine notions and augments one’s spirit, socio-emotional states, academic performance, classroom participation, engagement, and interpersonal communication skills (Loreman, 2011). By the same token, this love-oriented teaching prevents and minimizes the danger of various negative educational pitfalls like stress, lack of interest, tension, demotivation, amotivation, shyness, hopelessness, anxiety, shame, boredom, and so forth. Moreover, in a review study, Barcelos and Coelho (2016) constructed six core components for a loving pedagogy, including *ethics*, *growth*, *care*, *respect*, *freedom*, and *dialogue*, which are all consistent with the tenets of PP and the affective turn in pedagogy. Additionally, they argued that endorsing students’ emotions and potentials and regarding language and love as two pivotal elements of condition and development will help teachers reflect love in their instructional practices more effectively. All these 15 components of loving pedagogy are integrated into Figure 1.

### The Practice of a Loving Pedagogy: Suggesting a Tentative Model

**Figure 2:**
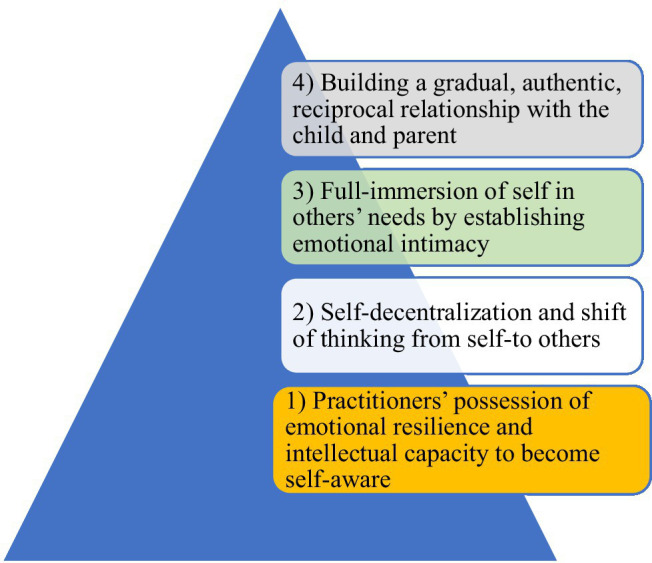
Steps in practicing professional love (Adopted from Page, 2018, p. 135).

As stated in this review article, the concept of “pedagogical love” or “a loving pedagogy” is really complicated as it involves many internal and external factors. Additionally, in the previous section, it was pinpointed that for an EFL teacher to implement a loving pedagogy, he/she needs knowledge, awareness, and skills considering various aspects of love represented through 15 core elements of a loving pedagogy (Figure 1). This multi-layered nature of such a pedagogy necessitates teachers and practitioners in L2 education to purely get engaged in/with discourse and praxis of love as the hallmark of a teaching career. Although research has clarified the definitions and benefits of love in education, proposing a practical framework to implement a loving pedagogy has been left uncharted, to date. Trying to take one of the first steps in this regard, the present study draws on the influential model proposed by Page (2018) and suggests a model for practicing professional love in SLA. This model was chosen because it was the first and the only tentative model of applying love in educational contexts. More particularly, the model of Page (2018) had four steps: (1) practitioners’ possession of emotional resilience and intellectual capacity to become self-aware; (2) self-decentralization and shift of thinking from self-to others; (3) full-immersion of self in others’ needs by establishing emotional intimacy; and (4) building a gradual, authentic, reciprocal relationship with the child and parent (Figure 2).

**Figure 3:**
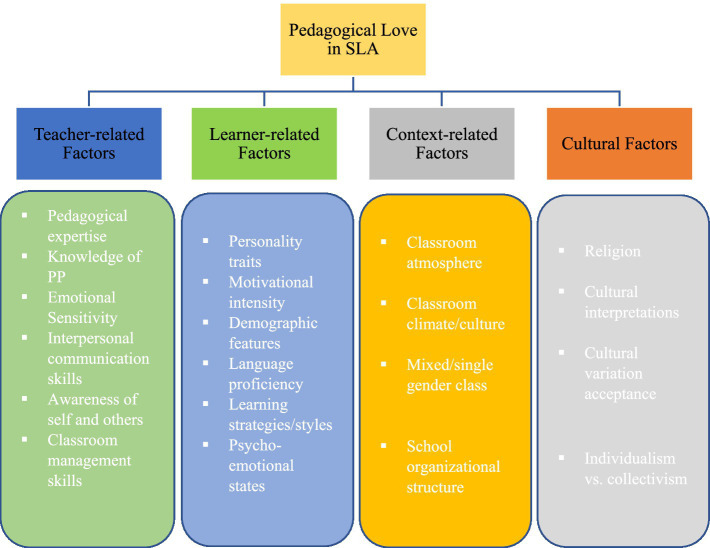
A model for the implementation of a pedagogy of love in second language acquisition (SLA).

Although this model, as one of the first propositions for applying love in education, is really appreciated and welcomed among educators, it should not be taken for granted and implemented in SLA without making revisions and extensions to it as the model was originally developed for early education. In the context of SLA, which has its idiosyncrasies and unique characteristics, the implementation of a loving pedagogy by teachers requires a new model going well with the discipline. Having this in mind, we would make an effort to revise the model of Page (2018) to make it appropriate in SLA research and practice. As illustrated, the model of Page (2018) put all eggs in teachers’/practitioners’ basket and turned a blind eye to other influential factors. Hence, it is advisable to design a model in which all factors determining an effective implementation of a loving pedagogy are included. Based on our model, in addition to teacher-related factors, SLA practitioners can take learner-related factors, context-related factors, and cultural issues into consideration when they wish to employ a love-based education. Additionally, a particular place can be allocated to PP, positive emotions, and other similar constructs in such a model (Figure 3).

Based on this model, to implement and practice a loving pedagogy in EFL/ESL contexts, teachers and practitioners need to consider numerous factors and issues so that desired outcomes can appear as a result of a love-based education. Teachers should have emotional sensitivity toward others and their needs and be skillful in using interpersonal communication skills, such as clarity, credibility, and immediacy (Xie and Derakhshan, 2021) to establish an atmosphere full of love and engagement on the part of the learners. Moreover, learners also have a critical role in the feasibility and practicality of a loving pedagogy in that their language level, motivation, engagement, positive emotions, and demographic features (e.g., age, gender, and education) definitely affect their acceptance of a loving pedagogy. In EFL classrooms which are full of setbacks and adversities, teaching and learning should be driven toward learners’ emotions at different levels to generate academic success (Mercer, 2020). Another important area pertains to classroom culture/climate/atmosphere, which strongly determines the acceptance and practice of a loving pedagogy. In a detached and dispassionate context, it is unwise to expect teachers and learners to seek for self and others’ emotional needs and desires. Likewise, the organizational structure of academic centers and schools is very important in that it can pave the way for emotion-based instruction or decrease the efficacy of methodological attempts made by teachers and learners. Finally, to implement a loving pedagogy in an EFL/ESL context, teachers can examine students’ religious beliefs, (inter)cultural awareness, and their degree of acceptance of some appeared-to-be taboo terms. Moreover, they need to be cognizant of the fact that implementing a pedagogy of love in individualistic countries (West) is not the same as in collectivists (Asia) countries (Guess, 2004). Individualistic cultures rarely care about others’ emotions as the first priority. In contrast, collectivist countries that see everything from a group angle (Mesquita, 2001; Lim, 2016) seem to have more fertile grounds for spreading the seeds of a loving pedagogy. Aside from all these factors, it is essential to note that the concept of love flourished in PP, hence, it is of paramount importance to consider PP and its constructs when practicing a loving pedagogy. The model is by no means without flaws and calls for more complementary studies in order to provide a comprehensive picture of a pedagogy of love in SLA.

### Research and Loving Pedagogy

The available literature corroborates that positive emotions, love, and a loving pedagogy are influential in L2 education from different perspectives (Barcelos, 2020; Wang et al., 2021). These seminal studies indicated that a loving pedagogy instruction can bring about numerous positive outcomes for EFL students and teachers (Barcelos, 2020) including their improved motivation, agency, autonomy, interpersonal communication skills, academic engagement, classroom participation, performance, resilience, achievement, creativity, identity (re)construction, and so on. Furthermore, it has been empirically identified that loving pedagogy boosts students’ classroom enjoyment, engagement, perseverance, self-discipline, relational connection, excitement, empowerment, and perceived success (Gharabaghi, 2008; Määttä and Uusiautti, 2013; Vincent, 2016b; Pavelescu and Petrić, 2018; Zhao and Li, 2021).

In a similar manner, pedagogical love can decrease negative stressors like stress, anxiety, and lack of interest among L2 students. As researching a loving pedagogy is still in its infancy, and it has lately entered the research zone of SLA, the number of empirical studies is scanty but growing. As for their research designs and tools, most studies on love in L2 education have utilized questionnaires, written narratives, case studies, interviews, and observation (Johnson et al., 2017; Li and Rawal, 2018; Pavelescu and Petrić, 2018; Yin et al., 2019; Barcelos, 2020). In addition to the instruments we have suggested above, we believe that a loving pedagogy demands other research instruments, such as diaries, journals, portfolios, as well as novel designs, such as experimental designs, longitudinal research, and time-series designs to capture the intricacies and developmental trajectory of how and in what ways a loving pedagogy can occur best and produce desirable academic outcomes. One-shot, quantitative designs do not provide a vivid image of this broad trend in language education. Consequently, researchers are invited to run complementary studies using tools and designs of various nature to position an agenda on a loving pedagogy research in SLA.

### Implications and Future Directions

In our review, it was argued that a pedagogy of love can bring about numerous positive outcomes in academic contexts. This is dependent on its judicial and proper implementation using context-specific models and frameworks. It was also maintained that the practice of a loving pedagogy is a function of many factors internal and external to EFL teachers/students. Trying to propose a research and practical agenda on a loving pedagogy, the present paper took one of the first steps in this line of inquiry. Hence, it can have valuable implications for SLA practitioners, teachers, and researchers. More specifically, this study can be of help for SLA practitioners in that they can use its ideas and the suggested model to implement a loving pedagogy in their L2 contexts. EFL teachers can also benefit from this article in the sense that they can realize various elements constituting a loving pedagogy and their significance in learning. They can also boost students’ engagement and participation in the classroom by employing emotion-based instruction where a professional and pedagogical love paves the way for many aspects of L2 learning. Furthermore, researchers are another group that can take advantage of this study in that they can locate the existing gaps in this research zone and run complementary investigations to add to the relevant body of knowledge. For instance, they can use qualitative and longitudinal research designs to capture the intricacies of a loving pedagogy in SLA. Likewise, interested researchers can conduct cross-cultural studies on the association of a loving pedagogy and different PP constructs, especially the interpersonal communication skills. Correlational studies are also suggested, especially on the association of a loving pedagogy with work engagement/satisfaction. The effect of cultural variations is also an interesting venue for research in the future. Finally, future scholars are recommended to develop and revise the existing models of a loving pedagogy as they are by no means impeccable. Similarly, future researchers are recommended to implement such models in SLA, English for specific purposes (ESP), and English for academic purposes (EAP) contexts.

## Author Contributions

All authors listed here have made direct and substantial contribution to the current study and approved the final manuscript for its submission.

## Funding

This study was sponsored by Teacher Education Project of the Education Department of Henan Province, China, entitled “Using Online Learning Resources to Promote English as a Foreign Language Teachers’ Professional Development in the Chinese Middle School Context” (Grant No. 2022-JSJYYB-027).

## Conflict of Interest

The authors declare that the research was conducted in the absence of any commercial or financial relationships that could be construed as a potential conflict of interest.

## Publisher’s Note

All claims expressed in this article are solely those of the authors and do not necessarily represent those of their affiliated organizations, or those of the publisher, the editors and the reviewers. Any product that may be evaluated in this article, or claim that may be made by its manufacturer, is not guaranteed or endorsed by the publisher.
